# Analysis of the Prevalence and Associated Risk Factors of Tinnitus in Adults

**DOI:** 10.1371/journal.pone.0127578

**Published:** 2015-05-28

**Authors:** Hyung-Jong Kim, Hyo-Jeong Lee, Soo-Youn An, Songyong Sim, Bumjung Park, Si Whan Kim, Joong Seob Lee, Sung Kwang Hong, Hyo Geun Choi

**Affiliations:** 1 Department of Otorhinolaryngology-Head & Neck Surgery, Hallym University Sacred Heart Hospital, Anyang, Korea; 2 Department of Otorhinolaryngology-Head & Neck Surgery, Thyroid/Head & Neck Cancer Center of the Dongnam Institute of Radiological & Medical Sciences (DIRAMS), Busan, Korea; 3 Department of Statistics, Hallym University, Chuncheon, Korea; University of Science and Technology of China, CHINA

## Abstract

**Background:**

Tinnitus is a common condition in adults; however, the pathophysiology of tinnitus remains unclear, and no large population-based study has assessed the associated risk factors. The aim of this study was to analyze the prevalence and associated risk factors of tinnitus.

**Methods:**

We conducted a cross-sectional study using data from the Korea National Health and Nutrition Examination Survey, with 19,290 participants ranging in age from 20 to 98 years old, between 2009 and 2012. We investigated the prevalence of tinnitus using a questionnaire and analyzed various possible factors associated with tinnitus using simple and multiple logistic regression analysis with complex sampling.

**Results:**

The prevalence of tinnitus was 20.7%, and the rates of tinnitus associated with no discomfort, moderate annoyance, and severe annoyance were 69.2%, 27.9%, and 3.0%, respectively. The prevalence of tinnitus and the rates of annoying tinnitus increased with age. The adjusted odds ratio (AOR) of tinnitus was higher for females, those with a smoking history, those reporting less sleep (≤ 6 h), those with more stress, those in smaller households, those with a history of hyperlipidemia osteoarthritis, rheumatoid arthritis, asthma, depression, thyroid disease, an abnormal tympanic membrane, unilateral hearing loss, bilateral hearing loss, noise exposure from earphones, noise exposure at the workplace, noise exposure outside the workplace, and brief noise exposure. Additionally, unemployed individuals and soldiers had higher AORs for tinnitus. The AOR of annoying tinnitus increased with age, stress, history of hyperlipidemia, unilateral hearing loss, and bilateral hearing loss.

**Conclusions:**

Tinnitus is very common in the general population and is associated with gender, smoking, stress, sleep, hearing loss, hyperlipidemia, osteoarthritis, rheumatoid arthritis, asthma, depression, and thyroid disease history.

## Introduction

Tinnitus is defined as the perception of sound in the absence of an external or internal source. The prevalence of tinnitus has been reported to range from 6.6% to 18.6% [1–5], and it increases to 30% in those aged 55 years and older [6]. Despite this high prevalence, only approximately one quarter of adults with tinnitus seek medical help [7]. Therefore, small hospital-based studies cannot provide high-quality information regarding tinnitus-related factors.

The associations of hearing loss, noise exposure, stress, and depression with tinnitus are clear [8–12], whereas the roles of sex, alcohol consumption, smoking status, educational level, and income level differ among studies [13–19]. A history of arthritis was suggested to be associated with tinnitus [8, 15]. Cardiovascular disease risk factors, such as high body mass index (BMI), hypertension, diabetes mellitus, cerebral stroke, angina, or myocardial infarction, have been analyzed as possible risk factors of tinnitus in other studies [8, 9, 20, 21]; however, their associations with tinnitus are controversial. Few studies have evaluated a relationship between occupation and tinnitus [22, 23]. Only one study reported asthma as a related factor of tinnitus [17]. Tinnitus has been reported in some thyroid disease patients [10, 20, 24]; however, the association of thyroid disease with tinnitus has never been analyzed in a large population-based study. This study focused on these gaps in previous studies.

One study examined the prevalence of tinnitus in the general population of Korea, but the relationships between various factors and tinnitus have not been evaluated [25]. To our knowledge, this study is the largest to analyze the prevalence of tinnitus in the Korean population. We classified the risk factors of tinnitus in adults into the following four broad types: personal, socioeconomic, disease-related, and otological factors. This study evaluated the association of each factor with tinnitus.

## Materials and Methods

### Study Population and Data Collection

This study was approved by the Institutional Review Board of the Thyroid/Head & Neck Cancer Center of the Dongnam Institute of Radiological & Medical Sciences (DIRHAM’S IRB No. D-1401-002-002). Written informed consent was obtained from all participants prior to the survey.

This study is a cross-sectional study using data from the Korea National Health and Nutrition Examination Survey. The study covers one nation using statistical methods based on designed sampling and adjusted weighted values. The fourth and fifth Korea National Health and Nutrition Examination Surveys (KNHANES) from 2009, 2010, 2011, and 2012 were analyzed. The data were collected by the Centers for Disease Control and Prevention of Korea. Each year, a panel selected 192 enumeration districts and 20 households in each district for proper sampling to reflect the entire Korean population. These data represent the civilian, non-institutionalized South Korean population using stratified, multistage clustered sampling based on National Census Data by the National Statistical Office. The sampling was weighted by statisticians by adjusting the post-stratification, non-response rate and extreme values.

Among the 36,067 participants, we excluded the following participants: participants less than 20 years of age (8,875 participants); participants who failed or refused a pure-tone audiometry test (7,097 participants); and participants who reported incomplete data regarding height, weight, smoking, alcohol habits, sleep, stress level, educational level, income level, number of people in the household, occupation, or history of hypertension, diabetes mellitus, hyperlipidemia, cerebral stroke, angina or myocardial infarction, osteoarthritis, rheumatoid arthritis, pulmonary tuberculosis, asthma, atopic dermatitis, depression, thyroid disease, and tympanic membrane (805 participants). Ultimately, 19,290 participants (8,244 male, 11,046 female) were included in this study. The survey participants ranged from 20 to 98 years of age. The mean age of the overall population was 45.49±0.21 years. Of 19,290 participants, noise exposure histories were available for 15,865 participants. After applying the weighted values recommended by KNHANES, the adjusted odds ratios (AORs) were estimated.

### Survey

The participants were asked if they had heard any ringing, buzzing, roaring, or hissing sounds without an external acoustic source in the past year. The response options were “Yes”, “No”, and “I cannot remember”. The 43 participants who answered “I cannot remember” were grouped with the participants who answered “No”. Then, the participants were asked about the severity of the tinnitus: “Do these sounds bother you?” The response options were “No”, “A little annoying”, and “Very annoying”. Factors including noise exposure history with earphone use, at the workplace (for more than 3 months), at a place other than the workplace, for more than 5 h per week, and brief noise exposure, such as noise from firearms and explosions, were surveyed.

Each participant’s age, sex, BMI (kg/m^2^), smoking status, alcohol consumption, amount of sleep per day, and stress level in daily life were categorized as personal factors in this study. Smoking status was divided into four groups according to current status: no smoking, quit more than 1 year ago, smoking but not every day, and smoking every day. The participants who quit smoking less than 1 year ago were categorized into the “smoking but not every day” or “smoking every day” group according to their previous habits. Alcohol consumption was divided into six groups: never in the past year, less than once a month, 1 time per month, 2–4 times per month, 2–3 times per week, and 4 or more times per week. Amount of sleep was divided into three groups: ≤ 6 h per day, 7–8 h per day, and ≥ 9 h per day. The participants were asked if they usually feel stress, and the stress level was divided into the following four groups: no stress, some stress, moderate stress, and severe stress.

To evaluate socioeconomic factors, the number of household members, education level, monthly income, and occupation were used. Education level was divided into five groups: middle school or less, graduated from high school, graduated from junior college, graduated from college, and graduated from graduate school. By dividing the household income by the square root of the number of household members, the monthly income level was divided into 4 quartiles: lowest, lower middle, upper middle, and highest. Occupation was classified into 10 standard Korean occupations: manager; expert; specialist; clerk; service worker; salesperson; farmer or fisherman; technician, mechanic, production worker, or engineer; laborer; and soldier. Unemployed participants comprised an eleventh group.

The participants were asked about their histories of other comorbidities, such as hypertension, diabetes mellitus, hyperlipidemia, cerebral stroke, angina, myocardial infarction, osteoarthritis, rheumatoid arthritis, pulmonary tuberculosis, asthma, atopic dermatitis, depression, and thyroid disease, and those who reported a history of any of these diseases, as diagnosed by a medical doctor, were recorded as positive.

In this study, trained otorhinolaryngologists examined all of the tympanic membranes (TMs) and categorized the right and left TMs into the following three groups: normal, abnormal, and could not examine. The subjects were then grouped as either having normal TMs or as having abnormal TMs. Pure-tone audiometry for threshold measurements was performed at 500, 1000, 2000, 3000, 4000, and 6000 Hz in both ears in a soundproof booth using an automatic audiometer (SA 203, Entomed, Sweden). Hearing loss was defined as a hearing loss exceeding an average of 25 dB on pure-tone audiometry at 500, 1000, 2000, and 4000 Hz [26]. Hearing loss was classified into the following three groups: normal, unilateral hearing loss, and bilateral hearing loss.

To analyze the association between the examined factors and the annoyance associated with tinnitus, we categorized the severity of tinnitus into two groups: no discomfort and annoying tinnitus (slightly annoying and very annoying). The OR of annoying tinnitus was calculated compared with tinnitus associated with no discomfort.

### Statistical Analysis

The associations between the risk factors and tinnitus were analyzed using simple and multiple logistic regression analysis with complex sampling. The association between the noise exposure history and tinnitus was calculated using multiple logistic regression analysis with complex sampling adjusting for age, sex, stress level, and hearing. To identify associations between the related factors and tinnitus annoyance, multiple logistic regression analysis with complex sampling was used. In the multiple logistic regression analysis, independent variables (except for age and sex) were selected by backward elimination. Two-tailed analyses were conducted, and *P*-values lower than 0.05 were considered to indicate significance. The adjusted odds ratio (AOR) and 95% confidence interval (CI) for tinnitus were calculated. All results are presented as weighted values. The results were analyzed statistically using SPSS ver. 21.0 (IBM, Armonk, NY, USA).

## Results

### Prevalence and Severity of Tinnitus

**Fig 1:**
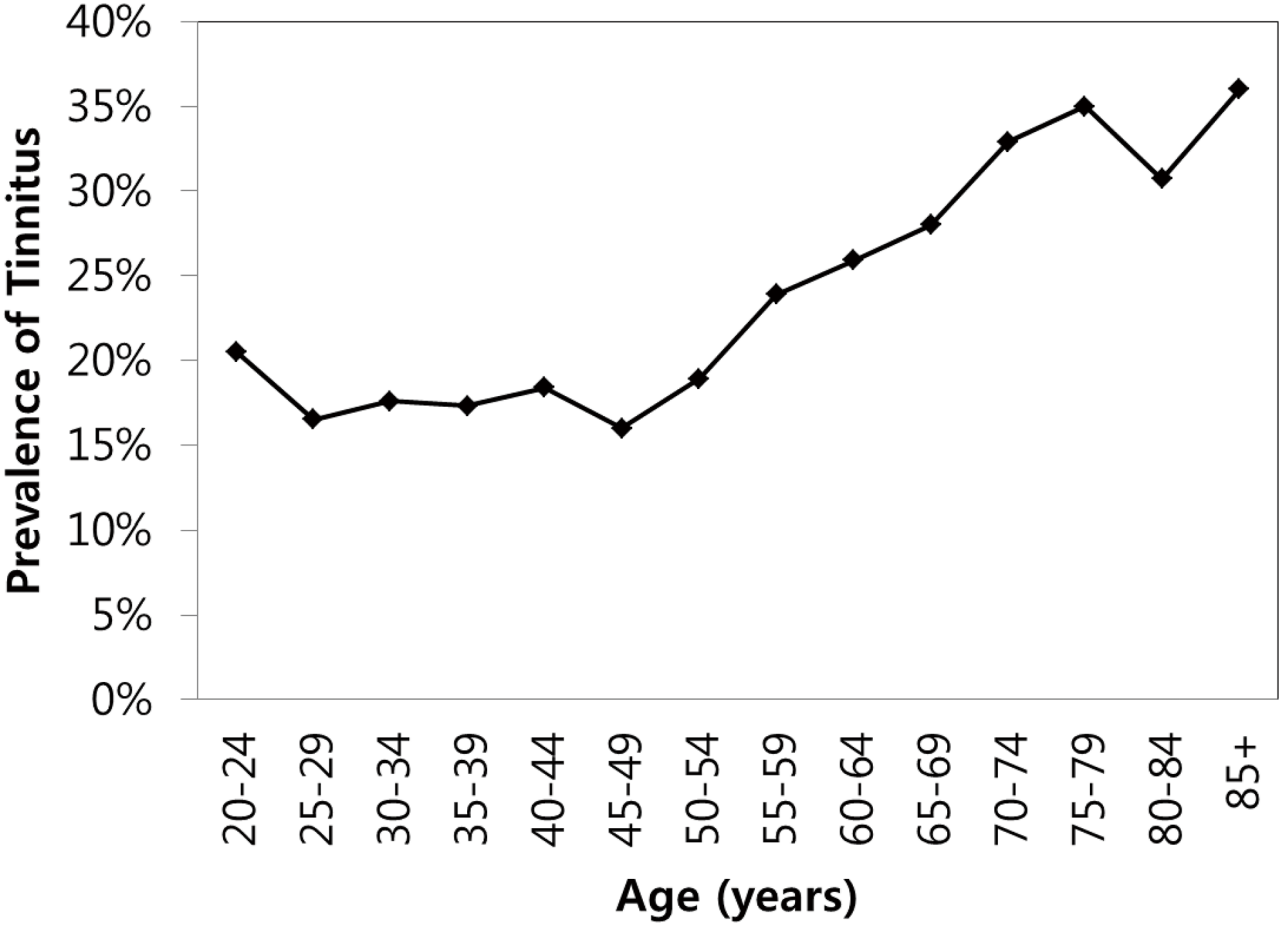
Prevalence of tinnitus in the Korean adult population.

Of the 19,290 subjects, 4,234 (20.7%, estimated rate) experienced tinnitus within the past year. In most of the subjects (2,800, 69.2%), the tinnitus caused no discomfort, while 27.9% (1,288) were slightly annoyed by the tinnitus, and only 3% (146) complained of severe discomfort due to tinnitus (Table 1). The prevalence of tinnitus increased with age and was < ~20% in subjects under 50 years of age. The highest prevalence was 36.0% in the 85+ age group, and the lowest prevalence was 16.0% in the 45–49 age group (Fig 1).

**Table 1:** Prevalence and Severity of Tinnitus.

	Rates	%^a^
Have you heard any ringing, buzzing, roaring, or hissing sounds without an external acoustic source in the past year?		
Yes	4,234/19,290	20.7 ± 0.4
No	15,056/19,290	79.3 ± 0.4
Do these sounds bother you?		
No	2,800/4,234	69.2 ± 1.1
A little annoying	1,288/4,234	27.9 ± 1.0
Very annoying	146/4,234	3.0 ± 0.3

^a^Estimated rate, adjusted with weight values

### Simple Logistic Regression Analysis of Risk Factors of Tinnitus

Aging (10 years) increased the OR of tinnitus 1.17-fold. Female gender was significantly associated with tinnitus (OR = 1.32). Neither BMI nor smoking was associated with tinnitus. Alcohol consumption was inversely associated with tinnitus (*drinking four or more times per week*, OR = 0.78, *drinking 2–3 times per week*, OR = 0.76; *drinking 2–4 times per month*, OR = 0.68; *drinking once per month*, OR = 0.74; *drinking less than once per month*, OR = 0.82). Compared with subjects who sleep ≤ 6 hours, subjects who sleep 7–8 hours had a lower OR (0.81). Stress increased the OR of tinnitus (*some stress*, OR = 0.97; *moderate stress*, OR = 1.34; *severe stress*, OR = 1.61).

The number of household members was negatively associated with tinnitus (OR = 0.89). Compared with subjects with a middle school education or less, a higher level of education was inversely associated with tinnitus (*high school*, OR = 0.63; *junior college*, OR = 0.54; *college*, OR = 0.59; *graduate school*, OR = 0.50). Higher income was negatively associated with tinnitus (Middle, low, OR = 0.68; Middle, high, OR = 0.58; Highest, OR = 0.55). Compared with the unemployed, some occupations were positively correlated with tinnitus. (*salesperson*, OR = 1.85; *mechanic*, *production worker*, *or engineer*, OR = 1.84; *laborer*, OR = 2.88; *soldier*, OR = 1.80).

A history of hypertension, diabetes mellitus, hyperlipidemia, cerebral stroke, angina or myocardial infarction, osteoarthritis, rheumatoid arthritis, pulmonary tuberculosis, asthma, depression, or thyroid disease was positively associated with tinnitus (*hypertension*, OR = 1.45; *diabetes mellitus*, OR = 1.47; *hyperlipidemia*, OR = 1.43; *cerebral stroke*, OR = 1.74; *angina or myocardial infarction*, OR = 1.78; *osteoarthritis*, OR = 1.83; *rheumatoid arthritis*, OR = 1.89; *pulmonary tuberculosis*, OR = 1.23; *asthma*, OR = 1.76; *depression*, OR = 2.02; *thyroid disease*, OR = 1.59).

Having an abnormal TM increased the OR of tinnitus (OR = 1.68). Unilateral hearing loss (> 25 dB) increased the OR of tinnitus 1.97-fold, and bilateral hearing loss increased the OR of tinnitus 3.01-fold (Table 2).

**Table 2:** Simple and multiple logistic regression analysis with complex sampling for tinnitus.

Related Factors		Simple			Multiple		
	%^a^	AOR	95% CI	P-value	AOR	95% CI	P-value
**Personal Factors**							
Age (10 years)		1.17	1.13–1.21	<0.001 ^b^	0.96	0.92–1.00	0.069
Sex				<0.001 ^b^			<0.001^b^
Male	49.7	1			1		
Female	50.3	1.32	1.21–1.43		1.33	1.16–1.53	
BMI (kg/m^2^)		0.99	0.98–1.00	0.081			
Current Smoking				0.396			0.035 ^b^
No smoking	53.4	1			1		
Quit (> 1year)	15.7	1.03	0.92–1.16		1.25	1.06–1.47	
Smoking, not everyday	5.1	0.93	0.76–1.13		1.12	0.90–1.40	
Smoking, everyday	25.8	0.92	0.82–1.03		1.21	1.04–1.41	
Alcohol consumption				<0.001 ^b^			
Never in a year	23.3	1					
< 1 times a month	18.5	0.82	0.72–0.93				
1 times a month	10.5	0.74	0.63–0.87				
2–4 times a month	24.3	0.68	0.60–0.77				
2–3 times a week	15.8	0.76	0.66–0.87				
≥ 4 times a week	7.7	0.78	0.65–0.93				
Sleep time				<0.001 ^b^			0.027 ^b^
≤ 6 Hour	40.3	1			1		
7–8 Hour	52.3	0.81	0.74–0.88		0.88	0.80–0.97	
≥ 9 Hour	7.5	1.04	0.89–1.23		0.97	0.82–1.15	
Stress				<0.001 ^b^			<0.001 ^b^
No	13.2	1			1		
Some	58.6	0.97	0.86–1.09		1.19	1.04–1.35	
Moderate	23.5	1.34	1.17–1.54		1.62	1.40–1.87	
Severe	4.7	1.61	1.30–2.00		1.74	1.39–2.19	
**Socioeconomic Factors**							
N of household		0.89	0.86–0.92	<0.001 ^b^	0.96	0.92–1.00	0.025 ^b^
Education level				<0.001 ^b^			
Uneducated, elementary school, middle school	27.4	1					
High school	30.9	0.63	0.56–0.70				
Junior college	14.2	0.54	0.46–0.62				
College	23.5	0.59	0.52–0.67				
Graduated school	3.9	0.50	0.39–0.64				
Monthly income				<0.001 ^b^			
Lowest	15.6	1					
Middle, low	26.7	0.68	0.60–0.77				
Middle, high	29.4	0.58	0.51–0.65				
Highest	28.2	0.55	0.48–0.62				
Occupation				<0.001 ^b^			0.008 ^b^
Manager	1.5	1.15	0.79–1.66		0.68	0.48–0.97	
Expert, Specialist	12.7	1.15	0.79–1.69		0.84	0.72–0.99	
Clerk	9.2	1.25	0.84–1.86		0.83	0.69–1.00	
Service worker	6.4	1.19	0.82–1.75		0.81	0.64–1.01	
salesperson	7.5	1.85	1.28–2.68		0.79	0.65–0.96	
Farmer, Fisher	6.5	1.26	0.85–1.87		0.98	0.81–1.19	
Technician	6.2	1.11	0.74–1.66		0.87	0.69–1.10	
Mechanics, Production worker, Engineer	5.8	1.84	1.27–2.67		0.82	0.65–1.05	
Laborer	8.4	2.88	1.39–5.94		1.05	0.88–1.24	
Soldier	0.3	1.80	1.39–5.94		2.22	1.10–4.46	
Unemployed	35.4	1			1		
**Disease Factors**							
Hypertension				<0.001 ^b^			
Yes	17.0	1.45	1.31–1.60				
No	83.0	1					
Diabetes mellitus				<0.001 ^b^			
Yes	6.4	1.47	1.26–1.71				
No	93.6	1					
Hyperlipidemia				<0.001 ^b^			0.014 ^b^
Yes	8.3	1.43	1.26–1.64		1.19	1.04–1.38	
No	91.7	1			1		
Cerebral stroke				<0.001 ^b^			
Yes	1.3	1.74	1.33–2.26				
No	98.7	1					
Angina or myocardial infarction				<0.001 ^b^			
Yes	1.7	1.78	1.42–2.22				
No	98.3	1					
Osteoarthritis				<0.001 ^b^			0.008 ^b^
Yes	8.2	1.83	1.62–2.07		1.22	1.05–1.41	
No	91.8	1			1		
Rheumatoid arthritis				<0.001 ^b^			0.040 ^b^
Yes	1.5	1.89	1.45–2.48		1.32	1.01–1.74	
No	98.5	1			1		
Pulmonary tuberculosis				0.055			
Yes	4.3	1.23	1.00–1.52				
No	95.7	1					
Asthma				<0.001 ^b^			0.002 ^b^
Yes	2.9	1.76	1.41–2.19		1.44	1.14–1.82	
No	97.1	1			1		
Atopic dermatitis				0.279			
Yes	2.8	1.16	0.89–1.51				
No	97.2	1					
Depression				<0.001 ^b^			<0.001 ^b^
Yes	3.6	2.02	1.65–2.48		1.55	1.24–1.93	
No	96.4	1			1		
Thyroid disease				<0.001 ^b^			0.002 ^b^
Yes	3.1	1.59	1.31–1.93		1.37	1.13–1.67	
No	96.9	1			1		
**Otological Factors**							
Tympanic membrane				<0.001 ^b^			0.002 ^b^
Normal, both	91.2	1			1		
Abnormal	8.8	1.68	1.47–1.92		1.25	1.08–1.45	
Hearing loss (> 25dB)				<0.001 ^b^			<0.001 ^b^
No	78.6	1			1		
Unilateral	9.7	1.97	1.72–2.26		1.87	1.62–2.16	
Bilateral	11.7	3.01	2.71–3.35		2.86	2.48–3.30	

^a^Estimated rate, adjusted with weight values

^b^Significance at P < 0.05

### Multiple Logistic Regression Analysis of Risk Factors of Tinnitus

Age and alcohol consumption were not associated with tinnitus. Compared to nonsmokers, participants who quit smoking more than 1 year ago and every day smokers had higher AORs (1.25, 1.21, respectively). Compared with subjects who sleep ≤ 6 hours, subjects who sleep 7–8 hours had lower ORs (0.88). Stress increased the AOR of tinnitus (*some stress*, AOR = 1.19; *moderate stress*, AOR = 1.62; *severe stress*, AOR = 1.74).

Educational level and monthly income were not associated with tinnitus in this analysis. The number of household members was negatively associated with tinnitus (AOR = 0.96). Compared with the unemployed, some occupations were negatively associated with tinnitus, with the exception of soldiers (*Manager*, AOR = 0.68; *expert*, *specialist*, AOR = 0.84; *salesperson*, AOR = 0.79). Only soldiers had a positive association with tinnitus (AOR = 2.22).

A history of hypertension, diabetes mellitus, cerebral stroke, angina or myocardial infarction, pulmonary tuberculosis, and atopic dermatitis were not linked with tinnitus. A history of hyperlipidemia, osteoarthritis, rheumatoid arthritis, asthma, depression, or thyroid disease was positively associated with tinnitus (*hyperlipidemia*, AOR = 1.19; *osteoarthritis*, AOR = 1.22; *rheumatoid arthritis*, AOR = 1.32; *asthma*, AOR = 1.44; *depression*, AOR = 1.55; *thyroid disease*, AOR = 1.37).

Having an abnormal TM was associated with tinnitus (AOR = 1.25). Unilateral hearing loss (> 25 dB) increased the AOR of tinnitus 1.87-fold, and bilateral hearing loss increased the AOR of tinnitus 2.86-fold. In this analysis, statistically insignificant factors were eliminated by backward elimination (Table 2).

### Multiple Logistic Regression Analysis of the Noise Exposure History

In this analysis, 15,865 participants who recorded their noise exposure history were analyzed. When age, sex, stress level, and hearing were adjusted, noise exposure with earphones, noise exposure in the workplace, noise exposure outside the workplace, and brief noise exposure increased the AORs for tinnitus (1.29, 1.28, 1.44, and 1.45, respectively) (Table 3).

**Table 3:** Multiple logistic regression analysis with complex sampling of noise exposure history for tinnitus.

Related Factors	%^a^	AOR	95% CI	P-value
Have you experienced loud sound with earphone use?				0.007 ^b^
Yes	11.9	1.29	1.07–1.56	
No	88.1	1		
Have you experienced loud sound more than 3 months at workplace?				0.001 ^b^
Yes	13.6	1.28	1.10–1.48	
No	86.4	1		
Have you experienced loud sound more than 5 hours a week other than workplace?				0.025 ^b^
Yes	2.5	1.44	1.05–1.98	
No	97.5	1		
Have you experienced loud sound such as firearms or explosions?				<0.001 ^b^
Yes	24.7	1.45	1.27–1.68	
No	75.3	1		

^a^Estimated rate, adjusted with weight values

^b^Significance at P < 0.05

### Multiple Logistic Regression Analysis of the Annoyance of Tinnitus

**Fig 2:**
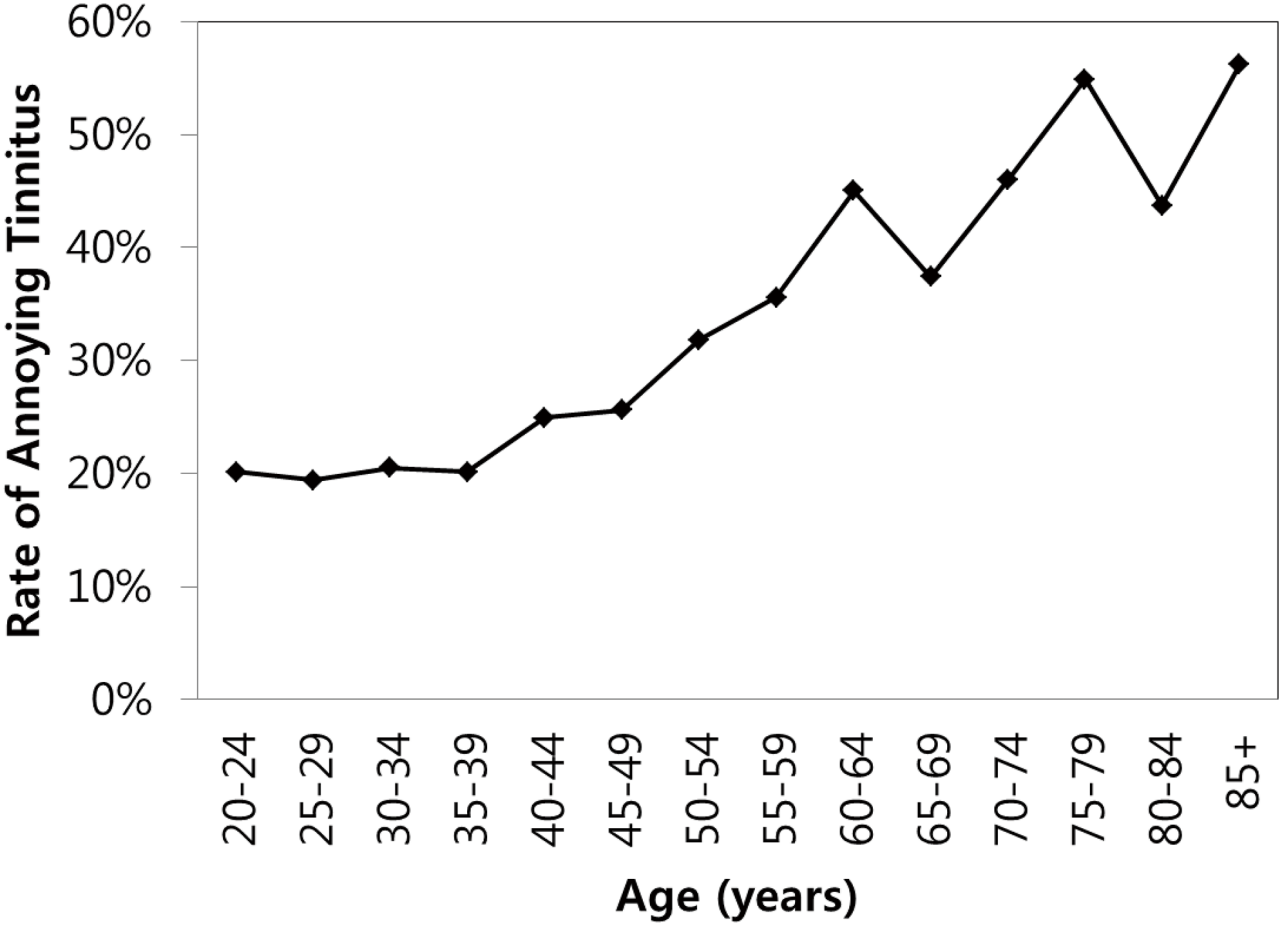
Rates of annoying tinnitus in the Korean adult population.

The rate of tinnitus described as annoying among the subjects who experienced tinnitus was 30.9%. This proportion increased with age, and the rate was > 30% for subjects over 50 years of age (Fig 2).

The annoyance associated with tinnitus was analyzed in 4,234 participants who experienced tinnitus. Aging (10 years) increased the AOR of annoying tinnitus 1.13-fold. Stress increased the AOR of annoying tinnitus (*some stress*, AOR = 1.18; *moderate stress*, AOR = 1.64; *severe stress*, AOR = 1.95). A history of hyperlipidemia was positively associated with annoying tinnitus (AOR = 1.52). Unilateral hearing loss (> 25 dB) increased the AOR of annoying tinnitus 1.88-fold, and bilateral hearing loss increased the AOR of annoying tinnitus 2.50-fold (Table 4). Because of the statistical insignificance, other factors were eliminated by backward elimination in this analysis.

**Table 4:** Multiple logistic regression analysis with complex sampling for tinnitus annoyance.

Related Factors	%^a^	AOR	95% CI	P-value
Age (10 years)		1.13	1.08–1.25	<0.001 ^b^
Sex				0.709
Male	44.3	1		
Female	55.7			
Stress				<0.001 ^b^
No	12.4	1		
Some	53.5	1.18	0.91–1.55	
Moderate	27.7	1.64	1.24–2.18	
Severe	6.3	1.95	1.30–2.90	
Hyperlipidemia				0.002 ^b^
Yes	10.6	1.52	1.17–1.97	
No	89.4	1		
Hearing loss (> 40dB)				<0.001 ^b^
No	64.9	1		
Unilateral	13.5	1.88	1.43–2.48	
Bilateral	21.6	2.50	1.98–3.16	

^a^Estimated rate, adjusted with weight values

^b^Significance at P < 0.05

## Discussion

We analyzed the ORs of tinnitus for various factors related to daily life. Some factors can reduce the likeliness of experiencing tinnitus, while others can increase the likeliness of experiencing tinnitus. Several of our results were in agreement with previous reports, whereas others were inconsistent with previous studies. Although we analyzed various factors, this study had the limitation of being cross-sectional; therefore, we could not rule out the possibility of reverse causality. For example, stress might be the cause of tinnitus, whereas tinnitus could also provoke stress. Therefore, our results should be interpreted carefully.

### Prevalence and Severity of Tinnitus

The prevalence of tinnitus was 20.7% (subjects older than 19 years who reported experiencing any tinnitus) in this study, which is considerably higher than in other population-based studies conducted in Asia; *e*.*g*., 11.9% in Japan (subjects 45 through 79 years old who experienced tinnitus for longer than 5 minutes) and 14.5% in China (subjects older than 10 years old who experienced tinnitus for longer than 5 minutes) [17, 27]. Our results were similar to those from England (18.4% of subjects 40 to 69 years old who experienced tinnitus for longer than 5 minutes) [28] and lower than reports from the US (25.3% of subjects older than 20 years old experienced any tinnitus) [9]. Because the age groups surveyed were not the same, it was difficult to compare the prevalence between studies. The definition of tinnitus also differed among studies. Participants who had temporary tinnitus were not excluded in this study, and the lack of definition of permanent tinnitus may have caused this observed difference in prevalence.

The prevalence of tinnitus was 16.0–20.5% in those 20 to 54 years old; it increased sharply after 55 years and exceeded 30% after 70 years (Fig 1). These results are consistent with those of Hoffman et al., who reported that the prevalence of tinnitus increases with age and plateaus at 60–69 or 70–79 years [29]. In our study, 3% of the subjects experienced severe discomfort from tinnitus, which is similar to other studies [17, 30] (Table 1).

### Associated Factors of Tinnitus

Hearing loss and noise exposure show a clear relationship with tinnitus [8, 9, 31]. Hearing loss above 25 dB increased the AOR of tinnitus in this study. In accordance with a previous report [32], bilateral hearing loss had a higher AOR than unilateral hearing loss.

The AORs for tinnitus differed among occupations [23]. Noise exposure and stress in the workplace affect the prevalence of tinnitus [23]. In accordance with previous studies [19, 33], the unemployed had a higher AOR for tinnitus than did workers. In our study, soldiers had the highest AOR for tinnitus. Soldiers are likely to be exposed to brief noises, such as the discharge of firearms, more often. This high AOR in soldiers reaffirms our finding that brief noise exposure is positively associated with tinnitus.

Workplace noise is a predictor of tinnitus [10, 12]. However, this was not the case in studies that examined an entire population instead of a “blue collar” group or those that adjusted for hearing level [21, 22]. Our results also show that the prevalence of tinnitus in “blue collar” populations, such as farmers, fishermen, mechanics, production workers, engineers, and laborers, was not different from its prevalence in the unemployed when adjusting for hearing level.

Stress is a well-known factor associated with tinnitus [11]. In accordance with previous studies [34], the greater the stress level, the greater the possibility of tinnitus.

Depression is closely associated with tinnitus [12, 35, 36]. Moreover, tinnitus can promote depression [37], and our results are in agreement with these findings.

### Unclear Factors of Tinnitus

Aging has been reported to be associated with tinnitus in many studies [13, 34]. We also found a higher prevalence of tinnitus in the elderly, and age was positively associated with tinnitus in a simple logistic regression analysis. However, we did not find a relationship between age and tinnitus in the multiple logistic regression analysis. We believe that hearing loss may act as the confounder between these factors.

Female gender was associated with tinnitus in this study. These results differ from those of several studies that reported a higher prevalence of tinnitus in males because of increased noise exposure [29, 34, 38]. However, other studies have found no association between gender and tinnitus when adjusting for other factors [8, 17, 32]. The discrepancies among these results may be due to differing social effects, such as the amount of noise exposure or stress exposure associated with gender in different countries.

One study reported that obesity decreases the OR of tinnitus [15], and another reported that the OR of tinnitus was higher in individuals with BMIs ≥ 30 kg/m^2^ [9]. However, we did not find a significant relationship between BMI and tinnitus in our simple and multiple logistic regression analyses.

Sleep disturbance due to tinnitus is a well-known phenomenon [5, 32, 36]. In the simple and multiple logistic regression analyses, 7–8 hours of sleep was negatively associated with tinnitus.

Smoking is positively associated with tinnitus in some studies [8, 15]. It has been suggested that smoking causes oxidative damage to the inner ear [39, 40]. Consistent with previous reports [8, 15], we found a positive association between smoking and tinnitus in the multiple logistic regression analysis.

Alcohol consumption has an inverse relationship with tinnitus in some studies. [8, 15]. It has been postulated that drinking alcohol has protective effects on the microvascular health of the cochlea [41]. However, in several studies, no association was found between alcohol consumption and tinnitus [16, 18, 42]. When adjusting for stress and otological factors, we could not find an association between alcohol consumption and tinnitus.

Several studies have reported that there is no association between education level and tinnitus [16, 42], while others have reported an inverse relationship [17, 43]. It has been suggested that less-educated people present greater emotional/cognitive impairment compared with in more-educated people [43]. In our study, education level was negatively associated with tinnitus in the simple logistic regression analysis. However, no relationship was found in the multiple logistic regression analysis. The association between tinnitus and income level is controversial [18, 19], and we did not find a relationship between income level and tinnitus through multiple logistic regression analysis. We suggest that the lower OR in groups with higher educational levels and incomes may be influenced by stress level, hearing loss, and occupational factors, as indicated by simple logistic regression analysis.

Hypertension, diabetes mellitus, cerebral stroke, angina, and myocardial infarction have been proposed as possible cardiovascular risk factors of tinnitus [8, 9, 20]. However, the associations of these factors with tinnitus are controversial [8, 15–18, 20, 42], and we did not find associations between these factors and tinnitus through multiple logistic regression analysis.

A recent study reported that the treatment of hyperlipidemia improves tinnitus [44, 45], in that high serum cholesterol has a negative impact on the inner ear, and elevated serum cholesterol has been proposed as a cofactor of hearing loss [46]. We found that a history of hyperlipidemia was positively associated with tinnitus through multiple logistic regression analysis.

Our study found high AORs for osteoarthritis and rheumatoid arthritis. Rheumatoid arthritis is an autoimmune disease, whereas osteoarthritis is a degenerative disease. Autoimmune disease has been reported as a related factor for tinnitus [20], as has a history of any arthritis [8, 15] because non-steroidal anti-inflammatory drugs (NSAIDs) prescribed for arthritis can affect tinnitus. However, we could not evaluate the history of arthritis medication in this study. Tinnitus has been reported as a side effect of NSAID use in osteoarthritis or rheumatoid arthritis [47, 48]. In addition, a higher prevalence of tinnitus in arthritis patients has been reported, regardless of medication use [42].

One study reported that asthma was an associated risk factor for tinnitus [17], which is consistent with our results. The inflammatory mediators of asthma are also involved in the pathogenesis of cardiovascular disease [49], and they may induce tinnitus by affecting the vasculature of the inner ear.

A history of thyroid disease was significantly associated with tinnitus. This factor is ambiguous and includes a broad range of thyroid diseases. However, tinnitus has been reported in various thyroid diseases, including autoimmune thyroiditis, hypothyroidism, and hyperthyroidism [10, 20, 24]. In our study, this association was significant and suggests that there is a relationship between tinnitus and thyroid disease.

A history of middle ear infection is associated with tinnitus [8, 12]. In our study, an abnormal TM resulted in a higher OR in both the simple and multiple logistic regression analyses.

### Annoyance of Tinnitus

Among the subjects who reported tinnitus, the rate of discomfort associated with tinnitus was highest in the elderly group (Fig 2), and age increased the AOR of annoying tinnitus. Adaptation plays an important role in tinnitus annoyance [50]. It is possible that older individuals have more difficulty adapting to tinnitus. The difficulty of habituation is influenced by psychological factors [51], and stress increased the AOR of annoying tinnitus in this study. It could be inferred that participants that experience higher levels of stress fail to habituate to tinnitus. In contrast to a previous study [52], gender was not associated with tinnitus annoyance in this study. Consistent with previous studies [52, 53], hearing loss was associated with tinnitus annoyance.

## Conclusions

In summary, female gender, stress, unemployment, being a soldier, hyperlipidemia, osteoarthritis, rheumatoid arthritis, asthma, depression, thyroid disease, hearing loss, noise exposure with earphones, noise exposure at the workplace, noise exposure at locations other than the workplace, and brief noise exposure are associated with tinnitus. This large population-based study provides reliable information regarding tinnitus associated factors and improves our understanding of tinnitus
